# The role of the pharmacist in the selection and use of over-the-counter proton-pump inhibitors

**DOI:** 10.1007/s11096-015-0150-z

**Published:** 2015-06-23

**Authors:** Helen F. Boardman, Gordon Heeley

**Affiliations:** School of Pharmacy, University of Nottingham, Nottingham, NG7 2RD UK; Lloydspharmacy, 113 Sneinton Road, Nottingham, NG2 4QL UK

**Keywords:** Counselling, Esomeprazole, Gastro-oesophageal reflux, Omeprazole, Over-the-counter therapy, Pharmacists, Proton-pump inhibitors

## Abstract

*Background* Heartburn and other symptoms of gastro-oesophageal reflux occur in ~30 % of survey respondents in multiple countries worldwide. Heartburn and acid regurgitation are common complaints in the pharmacy, where patients frequently seek relief through medication and advice. The growing number of proton-pump inhibitors available in the over-the-counter setting provides an efficacious choice to patients experiencing frequent heartburn. Pharmacists can assist patients in their treatment decisions whilst inquiring about alarm symptoms that should prompt a physician referral. *Aim of the review* Provide pharmacists with a review of current clinical research and expert guidelines on use of over-the-counter proton-pump inhibitors. *Methods* This narrative review was conducted to identify publications relevant to the following themes: overview of available treatments for frequent episodes of heartburn/acid regurgitation; treatment algorithms providing guidance on when to use over-the-counter proton-pump inhibitors; and the role of the pharmacist in the use of over-the-counter proton-pump inhibitors. *Results* Frequent symptoms of acid reflux, such as heartburn and acid regurgitation, can interfere substantially with daily life activities. Proton-pump inhibitors are the most efficacious treatment for frequent reflux symptoms and are recommended as an appropriate initial treatment in uncomplicated cases. Proton-pump inhibitors have varying pharmacokinetics and pharmacodynamics across the class; 20 mg esomeprazole has higher bioavailability and exposure than over-the-counter omeprazole, for example. However, differences in clinical efficacy for symptom relief have not been demonstrated. The safety and tolerability of proton-pump inhibitors have been well established in clinical trial and post-marketing settings, and use of a short regimen is associated with a very low likelihood of missing a more serious condition. Pharmacists can assist patients with accurate self-diagnosis by asking short, simple questions to characterize the nature, severity, and frequency of symptoms. Additionally, pharmacists can inquire about alarm symptoms that should prompt referral to a physician. Pharmacists should inform those patients for whom over-the-counter proton-pump inhibitors are appropriate on their proper use. *Conclusion* Over-the-counter proton-pump inhibitors have a valuable role in the treatment of frequent heartburn. Pharmacists have the opportunity to guide patients through selection of the best treatment option for their symptoms.

## Impacts on practice

- Familiarity with the range of products available to relieve symptoms of acid reflux, such as heartburn and acid regurgitation, and the evidence supporting their use in an over-the-counter setting will facilitate the pharmacist’s ability to guide appropriate selection and use of treatment.
- The evidence shows that proton-pump inhibitors at doses approved for over-the-counter settings can be used safely and effectively in appropriate patients.

## Introduction

Gastro-oesophageal reflux is a common medical problem with a broad spectrum of symptoms with varying degrees of severity and frequency [1–5]. The reflux movement of gastric contents into the oesophagus can cause a range of symptoms, from hiccups and burping to pain and physical discomfort [6]. In some cases, tissue damage can occur, leading to complications including ulcerating lesions and, in approximately 5–10 % of reflux cases, metaplasia of the lower oesophagus (Barrett’s oesophagus) [7, 8]. For most patients, visits to pharmacists and physicians are prompted when heartburn and acid regurgitation disrupt daily life [6].

There are an increasing number of options available to consumers for short-term management of reflux symptoms, which presents an opportunity for pharmacists to help guide appropriate selection of medications [6]. Guidelines such as those issued from the World Gastroenterology Organisation [9], the National Institute for Health and Care Excellence [10], and the American College of Gastroenterology [11] provide recommendations according to severity and frequency of symptoms, with changes to diet or lifestyle suggested for all patients and symptomatic use of antacids or alginates to provide relief for mild-to-moderate symptoms that occur intermittently. Moderate-to-severe symptoms with frequent recurrence should be approached with the intent to improve quality of life whilst preventing future episodes [7]. Because pharmacists are in the position to guide such selection, it is important for them to remain current with available products and the evidence supporting their use in an over-the-counter (OTC) setting [12].

## Aim of the review

The present review summarizes the clinical presentation of frequent reflux symptoms and available treatments. As many patients seek guidance from pharmacists on use of OTC medications [13], recommendations for identifying appropriate patients who could benefit from OTC proton-pump inhibitors (PPIs) and a summary of evidence on the class are provided. The role of the pharmacist in safe and appropriate use of OTC PPIs is discussed.

## Methods

A narrative review was conducted to identify publications relevant to the following themes:

- Frequent heartburn/acid regurgitation: overview of the condition and available treatments
- Treatment algorithm for frequent reflux symptoms: when to use OTC PPIs
- OTC PPIs: review of the class
- Role of the pharmacist in use of OTC PPIs

Literature searches were conducted using PubMed to identify English-language publications on the topics listed above from 1990 through 2014. Key search terms included, but were not limited to, heartburn; acid regurgitation; gastro-oesophageal reflux; non-prescription drugs; proton pump inhibitors; pharmacists; and professional role. Relevant literature was reviewed by the authors; information determined to be relevant to the selected themes of the review is summarized below. Additional relevant publications were identified via cross-referencing from the bibliographies of the selected articles. Primary data are supplemented with expert guidelines on best practice for the treatment of gastro-oesophageal reflux across the spectrum of severity and frequency. There are few pharmacokinetic and pharmacodynamic data available for OTC formulations of PPIs; we have presented available data for omeprazole 20 mg and esomeprazole 20 mg because these formulations are approved in the greatest number of countries and are available OTC in the largest markets.

## Results

### Frequent heartburn and acid regurgitation: overview of the condition and available treatments

Patients with gastro-oesophageal reflux report a range of symptoms that disrupt quality of life in terms of sleep, social functioning, overall activity levels, and work [1, 4, 6, 14, 15]. Reflux of stomach contents can cause burning sensations in the stomach, throat, oesophagus, and trachea, as well as pain in the stomach, middle of the chest, and in the back. Some patients describe regurgitation of food, an acid taste in the mouth, and halitosis; nausea or bloating; frequent and severe belching; or erosion of tooth enamel. Nocturnal symptoms, such as pain or coughing when lying down, can arouse patients and disturb sleep patterns.

Patients may describe having initially mild reflux-associated symptoms (such as hiccups, burping, mild heartburn), with the severity and frequency of symptoms increasing over time [6]. As the condition worsens, patients seek healthcare advice [6]. Reported triggers for consulting medical advice are symptom severity, interference with daily life, and questions about the potential for controlling symptoms [6].

A systematic review of population-based studies found that reflux symptoms were prevalent worldwide: in population-based studies mean prevalence rates (for periods up to 12 months) of at least weekly heartburn and/or acid regurgitation (or a physician’s diagnosis of gastro-oesophageal reflux) ranged from 18.1 to 27.8 % in North America, 8.8–25.9 % in Europe, 2.5–7.8 % in East Asia, 8.7–33.1 % in the Middle East, 11.6 % in Australia and 23.0 % in South America [16]. Frequent reflux is associated with typical symptoms, with 77 % of patients reporting heartburn, 63 % reporting acid regurgitation, and 41 % reporting both symptoms [4]. Frequent symptoms are more disruptive than intermittent episodes, although some patients delay seeking medical attention because they feel that the condition is not serious [4].

Symptomatic gastro-oesophageal reflux is treated with agents that counter the direct effects of the acid reflux on the oesophageal mucosa and/or medications that target gastric acid production associated with reflux, with different agents being more appropriate depending on the varying severity and frequency of episodes [4, 7, 17]. The initial recommendation for management is diet and lifestyle changes, such as weight loss and avoidance of foods or other substances that may trigger reflux [7, 11]. Antacids and alginates provide temporary, local relief of reflux symptoms and are recommended for intermittent symptoms of mild-to moderate intensity [7]. Data suggest that some alginate-containing compounds may be more effective than traditional antacids in post-prandial acid control [18]. Histamine receptor antagonists (H_2_RAs) block one source of the signalling cascade that induces gastric acid secretion [19]. H_2_RAs are administered twice daily, and over time, patients may develop a tolerance to these agents [7]. The most efficacious therapeutic class available for short-term treatment of acid reflux symptoms is PPIs [11, 20], which covalently bind the proton pumps that control the final step of gastric acid production [19]. The number of PPIs approved for OTC use is increasing [21, 22], and it is important for pharmacists to understand their place in therapy relative to other heartburn remedies. Unlike options that require multiple daily doses, PPIs are administered once daily [11] and provide a more complete and durable resolution of symptoms. In a head-to-head trial conducted in a general practice setting in France in patients with moderate heartburn with or without acid regurgitation occurring more than once weekly, 14-day treatment with an alginate (Gaviscon; Reckitt Benckiser Healthcare, Massy Cedex, France; 4 × 10 mL/day) was non-inferior to PPI treatment (omeprazole 20 mg/day) in time to onset but was less effective than omeprazole by day 7 in total heartburn-free days [23].

### Treatment algorithm for frequent reflux symptoms: when to use OTC PPIs

A short interview with a patient with symptoms consistent with acid reflux can identify the appropriate course of action: OTC medication or referral to physician for follow-up care [12]. Asking patients to describe their symptoms, including the frequency, nature, and severity of episodes, can confirm the correct course of action [12, 24]. It should be noted that the severity or frequency of symptoms is not necessarily a marker of underlying disease [25, 26]. As with all patients presenting with symptoms suggesting heartburn or acid regurgitation, follow-up questions should rule out the presence of alarm symptoms that should prompt a referral for further medical assessment (Table 1). Alarm features that could be identified in the pharmacy based on the patient’s responses include symptoms suggestive of cardiac-type chest pain, difficulty in or painful swallowing, recurrent bronchial symptoms/cough, hoarseness, signs/symptoms of gastrointestinal bleeding, and progressive unintentional weight loss [9]. Additionally, older patients who recently began experiencing reflux symptoms or patients who have a family history of gastrointestinal cancers should be referred to their physician [12, 24]. Pharmacists also should be aware that certain medications (e.g., nitrates and calcium antagonists) can predispose patients to reflux events and potentially precipitate or exacerbate reflux symptoms [27]. Suspicion of a drug-related cause of reflux symptoms would be a prompt for consultation with the prescribing physician.

**Table 1:** Questions to assist in identifying individuals who may benefit from over-the-counter proton-pump inhibitors versus those who should be referred [9, 12]

Inclusion questions	Exclusion questions
*Goal: Identify patient who may benefit from over*-*the*-*counter proton*-*pump inhibitor*	*Goal: Identify alarm symptoms that should prompt immediate referral*
What is the nature of the symptoms you are experiencing? How frequently are the symptoms occurring? Have you tried any lifestyle changes or medications that have made your symptoms better or worse?	When did the symptoms start? Have you experienced any unintentional weight loss, difficulties in or painful swallowing, recurrent cough, hoarseness/changes in voice, blood in faeces or vomit? Do you have a family history of gastric and/or oesophageal cancer?

Professional organizations and health authorities worldwide have similar guidance for the treatment of heartburn and other reflux symptoms [7, 10, 11]. Patients with intermittent reflux symptoms should introduce lifestyle and dietary changes and use antacids, alginates, or low-dose H_2_RAs on demand for symptom control [7]. Patients with frequent typical reflux symptoms should receive a course of PPIs to provide relief of symptoms prior to further diagnostic work-up [7, 11]. Endoscopy is not recommended for patients with typical symptoms unless they do not respond to PPI therapy [7, 11].

Because many current guidelines were developed before widespread OTC availability of PPIs, until recently there were few resources directed toward pharmacists to provide guidance. Consensus statements and expert algorithms for pharmacists are beginning to be developed, however, and as clinical guidelines are updated, the important role that pharmacists can and should play in the management of heartburn and acid regurgitation is becoming more apparent [12, 24].

### OTC PPIs: review of the class

#### Pharmacokinetics and pharmacodynamics

Multiple PPIs are currently available OTC, although the specific agents in the pharmacy may vary from country to country (Table 2) [21, 22]. PPIs are prodrugs that are converted into active compounds in the acidic environment of the gastric lumen [19]. The PPIs have distinct pharmacokinetic properties. As omeprazole is the most widely available in the pharmacy setting and esomeprazole is the most recently switched of the OTC PPIs [21, 22], we will present the pharmacokinetics of these agents at OTC doses. The reported exposure (area under the curve [AUC]) of these compounds after 5 days of administration ranges from 1.6 to 2.3 μmol h/L for omeprazole 20 mg through 2.8–4.2 μmol h/L for esomeprazole 20 mg [28–30]. The compound half-lives are similar, whilst the time to maximum serum concentration (t_max_) of omeprazole was longer than esomeprazole in one study [28–30].

**Table 2:** Over-the-counter proton-pump inhibitor availability by country [21, 22]

Drug	Year (region) of first OTC switch approval	Countries where approved for over-the-counter use^a^
Esomeprazole^b^	2013 (EU)	Austria, Belgium, Bulgaria, Croatia, Czech Republic, Denmark, Estonia, Finland, France, Germany, Greece, Hungary, Ireland, Italy, Lithuania, Norway, Poland, Portugal, Slovak Republic, Slovenia, Spain, Sweden, the Netherlands, UK, USA
Lansoprazole	2004 (Sweden)	*Australia,* Finland, *New Zealand*, Portugal, *Sweden*, USA
Omeprazole	1999 (Sweden)	Argentina, *Australia,* Austria, Belgium, Bulgaria, Canada, China, Czech Republic, Estonia, Finland, France, Germany, Hungary, Ireland, Italy, Lithuania, Mexico, *New Zealand*, Poland, Portugal, Slovak Republic, Slovenia, Spain, *Sweden*, Switzerland, *the Netherlands*, *UK*, USA
Pantoprazole^c^	2008 (Australia)	Argentina, *Australia*, Austria, Belgium, Bulgaria, Czech Republic, Denmark, Estonia, Finland, France, Germany, Greece, Hungary, *Ireland*, Italy, Lithuania, Mexico, *New Zealand*, *Norway*, Poland, Portugal, Slovak Republic, Slovenia, Spain, *Sweden*, Switzerland, *the Netherlands*, *UK*
Rabeprazole	2010 (Australia)	*Australia, UK*

^a^Italicized countries are those where the drug is approved for non-prescription sale in a pharmacy-only category

^b^Esomeprazole 20 mg was switched to non-prescription status under the centralised procedure in the entire European Union through a Decision of the European Commission dated 26 August 2013

^c^Pantoprazole 20 mg was switched to non-prescription status under the centralised procedure in the entire European Union through a Decision of the European Commission dated 12 June 2009

The bioavailability of single doses of omeprazole and esomeprazole has been reported to be 30–40 and 50 %, respectively [31–33]. After 5 days’ dosing, AUC was 80 % higher with esomeprazole 20 mg compared with omeprazole 20 mg [28]. A study of the pharmacokinetics of omeprazole in fed and fasting states demonstrated that exposure was somewhat lower after a breakfast compared with fasting conditions; however, this difference was not statistically significant (*P* = .2505) [34]. Administration after breakfast was associated with a longer t_max_ than whilst fasting (*P* = .0001) [34]. In a model of esomeprazole pharmacokinetics developed from 2 studies, the consistency between the AUC and the maximum concentration (C_max_) under fed and fasting states suggested that the effect of esomeprazole on gastric pH is not affected by food [35]. At present, dosing instructions recommend taking omeprazole in the morning, preferably without food, whilst esomeprazole may be taken any time with or without food [32, 33]. However, there is evidence suggesting that PPIs work best clinically when taken in the morning before breakfast [36].

Pharmacodynamic assessments of the effects of PPIs on gastric pH have been performed across multiple doses and time points [28, 37–39]. In studies of PPIs at OTC doses, esomeprazole 20 mg provided 11–13 h of gastric acid control [28, 37–39], with longer acid control (percentage of time intra-gastric pH >4) than pantoprazole 20 mg (*P* < .001 [37]; *P* < .0001 [39]), lansoprazole 15 mg (*P* = .0001 [38]; *P* = .026 [39]), and omeprazole 20 mg (*P* < .01) [28].

#### Drug–drug interactions

Clinically relevant drug–drug interactions with OTC PPIs are unlikely, but pharmacists should be aware of the potential issues given the important gatekeeper role they serve to prevent such situations. These interactions are well characterized in the product labelling [32, 33, 40, 41], but are summarized here for completeness.

Because PPIs are metabolized by hepatic cytochrome P450 (CYP) enzymes (CYP2C19 and CYP3A4), exposure may be influenced by inhibitors and inducers of these enzymes, and concomitant use with substrates of these enzymes could cause interactions. Studies have shown decreased exposure to clopidogrel, which is metabolized to its active metabolite via CYP2C19, when co-administered with PPIs including omeprazole and esomeprazole [32, 40]. Although the clinical relevance of this interaction is uncertain, concomitant use is discouraged as a precaution. PPIs decrease serum levels of the protease inhibitor atazanavir when used concomitantly, an interaction likely due to altered absorption from increased gastric pH; the combination is contraindicated with pantoprazole and not recommended with omeprazole and esomeprazole [32, 40, 41]. PPIs have the potential to alter the absorption of other drugs with gastric pH-dependent bioavailability (e.g., digoxin, ketoconazole) [32, 40, 41], and methotrexate levels have been reported to increase when given with PPIs [32, 41]. There have been a few isolated post-marketing cases in which PPIs resulted in changes to the international normalized ratio in patients taking anticoagulants (e.g., warfarin) [32, 41]. Although these interactions are well documented, they are unlikely to be clinically important for the majority of patients using OTC PPIs for a 14-day course.

#### Review of clinical data

The efficacy of OTC PPIs after short-term (14-day) regimens has been evaluated in clinical trials [42–46]. Due to differences in subject populations and study designs, these efficacy data cannot be directly compared. In parallel studies with each agent, over a 14-day treatment period, a regimen of PPIs at OTC doses was associated with similar improvements from baseline in the percentage of 24-hour heartburn-free days among subjects with frequent heartburn [42, 45, 46]. Nocturnal heartburn can be particularly problematic and has been shown to disrupt sleep patterns and impair overall quality of life [47]. A short course (14 days) of treatment with OTC PPIs has been shown to increase heartburn-free nights in subjects with frequent reflux symptoms at baseline [42, 45, 46]. A re-analysis of 14-day outcomes [48] from 2 previously published 4-week studies [49, 50] with esomeprazole 20 mg in individuals with frequent night-time heartburn and associated sleep disturbances demonstrated rapid resolution of heartburn episodes and sleep disturbances related to reflux symptoms.

Short-term courses of PPI therapy are well tolerated [12, 51–53]. Most adverse events in studies of 14-day treatment with OTC PPIs were mild and similar to placebo. The most commonly reported adverse events included headache (PPI: 1–3 % vs. placebo: <1–3 %) and mild gastrointestinal complaints such as diarrhoea (PPI: <1–3 % vs. placebo: <1–2 %), abdominal pain (PPI: <1–2 % vs. placebo: 1–2 %), and nausea (PPI: <1–2 % vs. placebo: 1 %) [42, 45, 46]. Rare occurrences of changes in laboratory values (such as elevated liver enzymes, low sodium or magnesium levels, and changes in white blood cell counts) have been reported in the overall clinical trial programs and post-marketing reports for PPIs [32, 40, 41, 53–55], although these changes have not been observed in 14-day studies with OTC PPIs [42, 45, 46].

With proper patient selection (including inclusion for frequent symptoms and exclusion for alarm symptoms), short-term use of PPIs has a low likelihood of masking a more serious condition or causing additional damage to the oesophagus [12, 56–59]. If a short course of PPIs is not effective, patients should then consult with a medical practitioner. Repeated courses of OTC PPIs (more than approximately 4 per year) should also prompt consultation.

A study performed in the United States to evaluate whether patients could self-diagnose for a frequent heartburn condition and follow use instructions, including the recommendation for seeking physician care if symptoms did not resolve, provided reassuring results [60]. Most study participants accurately self-diagnosed and were compliant with all instructions. Overall, 75 % of study participants consulted their physician about heartburn prior to, during, or after the study. Further, the instructions for use stated that a physician should be consulted if >14 doses were taken during the course of the study; a substantial proportion of participants (86 %) who took >14 doses consulted with a physician during the study [60].

These findings complement the long history of OTC use for anti-secretory drugs for heartburn treatment. Antacids and H_2_RAs have been available in pharmacies for decades [61]. At the time that H_2_RAs were under consideration for OTC availability, a decision analysis of patient behaviour and treatment efficacy concluded that OTC H_2_RAs would provide symptomatic relief to patients whilst not leading to an unsafe delay in seeking treatment for gastric cancer [61]. Present instructions for OTC PPIs limit use for short-term treatment of reflux symptoms to 14–28 days without consulting a physician [32, 40, 41, 55]. Patients should consult their physician if reflux symptoms recur immediately after a course of OTC PPIs [32, 40, 41, 55]. If symptoms recur after 4 months, a subsequent course of OTC PPIs can be initiated [9].

### Role of the pharmacist in the use of OTC PPIs

PPIs are widely acknowledged to be the most effective treatment for symptom relief of gastro-oesophageal reflux [11, 24]. Compared with other options, OTC PPIs have demonstrated benefits to symptom resolution and quality of life [24] and the practical advantage of more convenient once-daily dosing [11]. As more formerly prescription medications enter the OTC market, the role of the pharmacist in heartburn therapy has expanded, prompting the development of specific guidance for pharmacists [12, 24]. With their extensive knowledge of OTC drugs and their familiarity with their patients, pharmacists are ideally situated to assist in the selection of appropriate medication [12]. By asking the correct questions (Table 1) as outlined previously, pharmacists can confirm the presence of frequent reflux and perform an important surveillance function by identifying patients with alarm symptoms who should be referred to a physician [12]. These questions complement the available algorithms developed by expert gastroenterologists [12, 24]; however, adherence to a particular structure is not necessary.

Notably, these suggested questions are ones that should be posed to patients with any severity of symptoms, including those with intermittent symptoms that might be adequately addressed with antacids and alginates [7, 11]. It is important to remember that there is no correlation between frequency or intensity of the symptoms and the underlying severity of the condition [25].

Upon confirming the presence of reflux symptoms, pharmacists should review instructions with the patient to ensure the proper use of OTC PPIs. Patients should also be reminded that, unlike antacids, PPIs are not to be taken symptomatically [32]. Although the patients may feel better after a few days into the regimen, the mechanism of OTC PPIs is distinct from antacids, and consequently, the agents should be administered to their best advantage: antacids and alginates for local treatment of reflux symptoms; PPIs and H_2_RAs for the inhibition of gastric acid secretion [7, 19]. PPIs are administered once daily [32, 40, 41, 55]; treatment should be taken at the same time every day [40, 41, 55]. Currently available OTC PPIs are indicated for short-term regimens of 14–28 days [32, 40, 41, 55]. Although some patients take PPIs for months or years [62], a long-term treatment regimen should be monitored by a physician [32, 40, 41, 55].

The pharmacist should set the expectations of treatment with OTC PPIs. Patients may seek complete resolution of symptoms yet also anticipate immediate relief of symptoms [24]. Based on a meta-analysis of 18 studies with agents at different doses, approximately one-third of patients will experience relief of heartburn symptoms within a few days of starting a PPI regimen [24, 63], and approximately 55–80 % of patients experience first resolution/relief of heartburn symptoms within the first week of treatment, according to clinical trial data [32, 41]. Pharmacists should advise that lifestyle changes, including avoiding known triggers, are complementary to pharmaceutical treatments for gastro-oesophageal reflux and may increase the likelihood of treatment success [7, 11].

## Conclusion

Frequent reflux symptoms are not optimally managed with antacids or alginates [7, 11]. Guidelines consistently recommend a short-term course of PPIs for symptom relief in patients without alarm symptoms [7, 11]. Convenient, easy to use, and safe, OTC PPIs provide additional value to pharmacy patients with frequent reflux symptoms [7, 12]. Pharmacists are in the position to guide the selection of the best treatment by confirming the diagnosis, referring patients with alarm symptoms to physicians, and educating patients on the proper use of their OTC medication. Managing OTC PPI use in the pharmacy is a necessary and appropriate role for pharmacists.
